# Automated triage to a virtual lung nodule clinic using veye lung nodule volumetry provides a cost saving compared to standard care

**DOI:** 10.1093/bjro/tzag019

**Published:** 2026-09-25

**Authors:** Giles Dixon, Vruti Dattani, Vidan Masani, Pia Charters, Mary Jordan, Jonathan C L Rodrigues

**Affiliations:** Department of Respiratory Medicine, Royal United Hospitals Bath NHS Foundation Trust, Bath, BA13NG, United Kingdom; Department of Clinical & Biomedical Sciences, University of Exeter, Exeter EX2 2LU, United Kingdom; Department of Radiology, Royal United Hospitals Bath NHS Foundation Trust, Bath, BA13NG, United Kingdom; Department of Respiratory Medicine, Royal United Hospitals Bath NHS Foundation Trust, Bath, BA13NG, United Kingdom; Department of Radiology, Royal United Hospitals Bath NHS Foundation Trust, Bath, BA13NG, United Kingdom; York Health Economics Consortium, University of York, York, YO10 5DD, United Kingdom; Department of Radiology, Royal United Hospitals Bath NHS Foundation Trust, Bath, BA13NG, United Kingdom; Department of Health, University of Bath, Bath, BA27AY, United Kingdom

**Keywords:** computed tomography, pulmonary nodules, artificial intelligence

## Abstract

**Objectives:**

Pulmonary nodules are frequently identified on thoracic CT imaging, with management guided by established clinical pathways. Thoracic CT scans are reported by both specialist and general radiologists. This study evaluated discordance in pulmonary nodule identification and management between general and specialist radiologists and modeled automated triage to a virtual nodule clinic (VNC).

**Methods:**

All CT scans, including the thorax, performed over a 1-week period at a single UK center in March 2022 were identified and original reports reviewed. Oncology follow-up scans were excluded. Reports were categorized and compared with an independent second expert radiologist review. VNC triage pathways were modeled to select patients for second review by an expert radiologist. The pathways used combinations of original radiologist reports and automated nodule assessment (DeepHealth Ltd) to trigger VNC review. The cost implications of each pathway were calculated.

**Results:**

Among 243 CT scans, 98 (40%) original reports identified a pulmonary nodule. Expert review identified incorrect recommendations in 24/243 (10%) cases. Errors were significantly more frequent in reports issued by general radiologists compared with specialist thoracic radiologists (χ² ∼ 11.0, df = 2, *P *< .05). VNC triage pathways that selected cases for secondary review when automated nodule assessment failed to identify a nodule that was identified by the reporting radiologist resulted in the greatest cost savings per corrected nodule.

**Conclusion:**

Approximately 10% of thoracic CT scans contained errors in pulmonary nodule reporting. Cost-saving triage pathways combining non-specialist reporting with automated nodule assessment appear feasible and warrant prospective real-world validation.

**Advances in knowledge:**

This study demonstrates theoretical clinical implementation of semi-automated triage to a virtual nodule clinic and provides an evidence base for prospective study.

## Introduction

Lung nodules are small, round, or oval lesions ≤30 mm and are a common finding on thoracic computed tomography scans.^1^ Lung nodules are found in approximately 1/3 of chest CT scans but over 95% are benign.^2^^,^^3^ Accurate nodule assessment is required to ensure appropriate investigation and diagnosis whilst avoiding unnecessary follow-up.

In the United Kingdom, the management of lung nodules is governed by the British Thoracic Society guidelines, which recommend management and follow-up based on nodule morphology, size, and clinical parameters.^4^ This approach relies upon accurate visual interpretation of lung nodules by radiologists. The interobserver agreement for the identification of lung nodules is poor, and computer-aided detection has been highlighted as a potential solution.^5^

Deep learning detection and assessment of pulmonary nodules has several promising applications. These algorithms are trained on large lung cancer screening datasets to identify nodules and determine malignancy potential.^6^ These techniques can reproducibly and reliably identify pulmonary nodules and provide malignancy probabilities, which can help risk stratify patients.^7^^,^^8^

The impact of automated lung nodule detection on clinical pathways for patients with lung nodules has not been extensively reported. A recent NIHR Health Technology Assessment systematic review reported a lack of evidence on clinical outcomes or cost-effectiveness for artificial intelligence (AI)-assisted nodule detection.^9^ Furthermore, the impact of radiologist expertise in the assessment and management of lung nodules has not been reported.

We describe the incidence, evaluation, and management of pulmonary nodules in a real-world single-center CT cohort. We then modeled the impact of automated lung-nodule detection in triaging patients to a virtual nodule clinic (VNC), where a specialist thoracic radiologist could review cases and recommend management. We also conducted a cost-effectiveness analysis of the proposed AI-assisted pathways.

## Methods

### Ethics statement

The study was registered as a service evaluation with the host institution, who waived the need for patient consent.

### Study design

The study retrospectively assessed all CT scans, which included the thorax which were undertaken between March 21, 2022 and March 28, 2022 at a single site in the United Kingdom. The CT scans performed as oncology follow-up scans requested for malignancy surveillance were excluded as these are subject to alternative management pathways.

The original CT reports were analyzed and categorized according to indication, requesting specialty and expertise of radiologist (specialist/non-specialist or external radiologist). The reports were then categorized as to whether a nodule was identified and whether a BTS guideline-based recommendation for management was made.

All CT scans, then underwent second review, blinded to the original report, by a specialist thoracic radiologist to give a gold standard ground truth. Ground truth was then compared with the original radiologist report and discrepancies in reporting recorded. All discrepancies were classed as errors including missed nodules, incorrect suggested management plans and absence of suggested management plans.

### Automated nodule assessment

All scans then underwent retrospective analysis using Veye Lung Nodules (VLN) Version 2.0 (DeepHealth Ltd). In brief, VLN uses deep learning technology trained on over 45 000 chest CT scans with 40 000 radiologist annotations to identify nodules and provide an automated nodule volume.^10^ The cost per scan of VLN analysis was £4.00, which was included in the economic analysis.

### Hypothetical lung nodule management pathways

Three hypothetical lung nodule management pathways were then theoretically modeled (Figure 1). The models examined different pathways to identify patients who would benefit from review in a VNC by a specialist thoracic radiologist. A VLN volume of ≥80 mm^3^ was considered to be a positive automated identification of a nodule as per BTS guidelines for nodules that require follow-up.^4^

**Figure 1:**
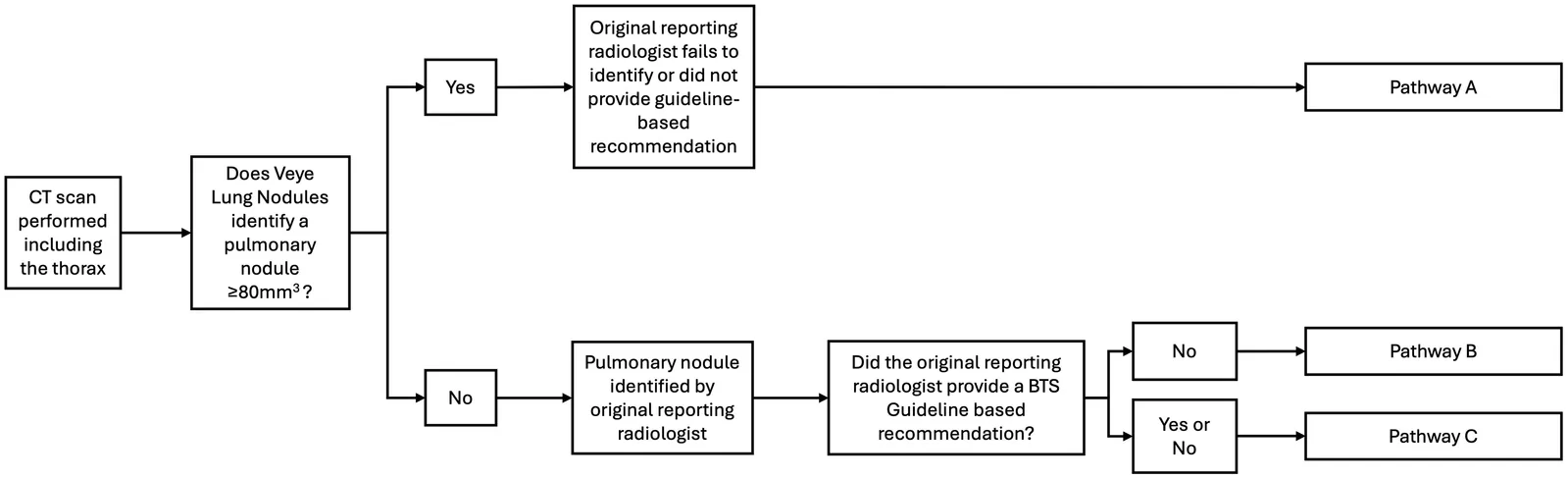
Proposed triage pathways A-C for entry of patients to virtual nodule clinic.

- Pathway A modeled a scenario whereby VLN identified a nodule, but the original radiologist failed to identify or did not provide a guideline-based recommendation.
- Pathway B modeled a scenario whereby VLN did not identify a nodule, but the original radiologist did identify the nodule without giving a guideline-based recommendation.
- Pathway C modeled a scenario whereby VLN did not identify a nodule, but the original radiologist did identify a nodule, with or without giving a guideline-based recommendation.

### Health economic analysis

The real-world consequences of incorrect recommendations were analyzed for patients after a minimum of 2 years from original CT scan. These consequences were obtained from electronic patient records and included additional unnecessary clinician or radiologist review, unnecessary MDT review, or unnecessary additional imaging.

Early economic evaluation of the three pathways was conducted by a health economist. A decision analytic model was developed in Microsoft Excel to explore the potential cost-effectiveness of each pathway from a UK healthcare provider perspective, over a 2-year time horizon.

Imaging test results and probabilities of patients moving through each pathway were sourced from audit data. Resource use associated with real-world consequences was derived from audit data and clinical expert opinion, with all costs extrapolated from routine published sources for the 2024/2025 cost year.^11^^,^^12^

## Results

### Analyzed CT scans

Between March 21 and March 28, 2022, 357 CT scans including the thorax were performed. 243/357 (68.1%) scans were included in the analysis (114/357 oncology follow-up scans excluded).

In Table 1, CT type, indication and original expertise of reporting radiologist for 243 CT scans performed between March 21, 2022 and March 28, 2022 excluding scans performed for oncology follow-up. Other included (but not limited to) indications such as tracheobronchomalacia, follow-up of non-nodule pulmonary finding and superior vena cava obstruction.

**Table 1.** CT type, indication and original expertise of reporting radiologist for 243 CT scans performed between 21/03/2022 and 28/03/2022 excluding scans performed for oncology follow up. Other included (but not limited to) indications such as tracheobronchomalacia, follow up of non-nodule pulmonary finding and superior vena cava obstruction.

**Type of CT scan**	CT thorax (non-contrast)	69
	CT thorax (contrast)	28
	CTPA	59
	High-resolution CT thorax	19
	CT Chest/Abdomen/Pelvis with contrast	68
**Reason for request**	Urgent suspected cancer	65
	Pulmonary embolism	53
	Lung nodule follow-up	31
	Cough or shortness of breath	27
	Trauma	16
	Interstitial lung disease	9
	Follow-up of abnormal chest radiograph	8
	Sepsis	7
	Pulmonary hypertension	3
	Covid-19 infection follow-up	3
	Oesophageal perforation	2
	Follow-up of consolidation	2
	Other	17
**Expertise of reporting radiologist**	Specialist thoracic radiologist	46
	General radiologist (internal)	80
	External (outsourced) radiologist	116
	Unknown	1

The 98/243 (40%) of original reports identified a lung nodule. 53/98 (54%) original reports had a guideline-based recommendation with 45/98 (46%) having no guideline based recommendation.

### Ground truth comparison

When compared to ground-truth reporting 24/243 (10%) scans had an incorrect or missing management pathway suggested by the original report (Figure 2). Within these 14/243 (6%) included errors within the proposed management plan, 6/243 (2%) were an absence of management plan and 4/243 (2%) were missed nodules (Table 2).

**Figure 2:**
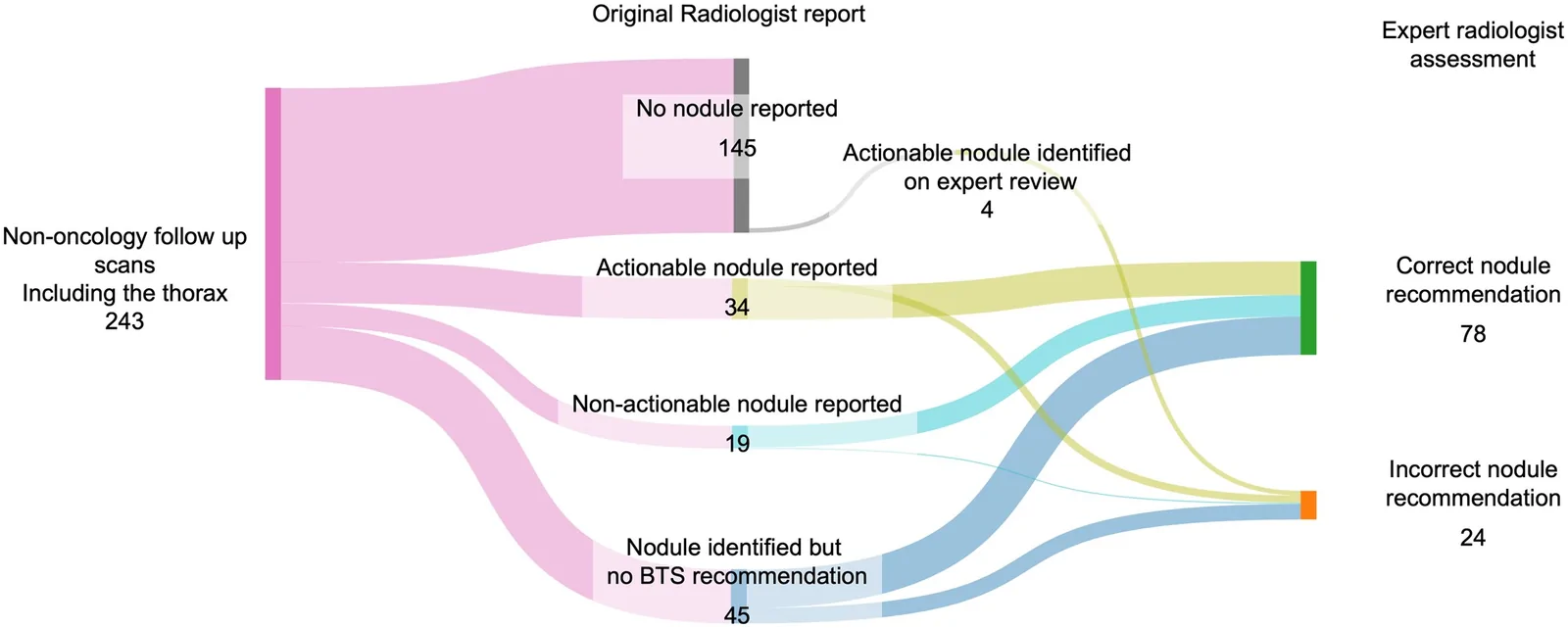
Sankey diagram comparing the original radiologist reports regarding pulmonary nodules of 243 non-oncology follow-up CT scans, including the thorax and secondary expert assessment.

**Table 2:** Summary of errors in original CT report when compared to ground truth review.

CT reporting error compared to ground truth	Frequency (*n*, % to nearest integer)
**No management plan suggested**	6 (2%)
**Incorrect timing of follow-up suggested**	5 (2%)
**Incorrect recommendation for follow-up being required**	4 (2%)
**Nodule not reported (missed)**	4 (2%)
**Incorrect recommendation for cessation of follow-up**	3 (1%)
**Incorrect recommendation for review in MDT**	2 (1%)

Abbreviation: MDT = Multi-disciplinary team.

Of the incorrect recommendations, 0/46 specialist radiologist reports were incorrect, 7/80 (9%) non-specialist (internal) radiologist reports were incorrect and 17/116 (15%) of external (outsourced) radiologist reports were incorrect. Fisher’s exact test identified a difference in error between groups (χ² ∼ 11.0, df = 2, *P *< .05). Pairwise comparison identified significant increase in error between both general radiologists (internal) and external (outsourced) radiologists and specialist thoracic radiologists (*P* = .047 and *P* = .003, respectively).

### VLN analysis

VLN was successful in 232/243 (96%) cases. At least one nodule ≥80 mm^3^ was identified in 80/243 scans (32%). Of the 152 scans with no nodules ≥80 mm^3^ identified 145/152 (96%) had no actionable nodule on ground-truth comparison. 6/152 (4%) had a nodule identified on ground-truth comparison requiring follow-up (3 ground glass nodules, 1 missed due to contouring failure and 2 not identified by VLN). These missed nodules were located in lingula (2), bilateral (1), right upper lobe (2), and right middle lobe adjacent to oblique fissure (1).

### Sensitivity/specificity of VLN analysis

Veye Lung Nodules had a sensitivity and specificity of 83.3% (45/54) and 81.5% (154/189) for the identification of actionable pulmonary nodules on ground truth analysis. The positive predictor value and negative predictor values were 56.3% (45/80) and 94.5% (154/163), respectively.

### Virtual nodule clinic pathways

Three models of triage into a hypothetical VNC were examined (Figure 1).

- Pathway A whereby VLN identifies a ≥80 mm^3^ nodule but was not identified by the original radiologist or a guideline-based recommendation was not given would result in 51/243 (21%) cases being reviewed in VNC with a correction in error for 12 incorrect reports.
- Pathway B whereby VLN does not identify a ≥80 mm^3^ nodule but original radiologist does without providing a guideline-based recommendation would lead to 14/243 (6%) cases reviewed in VNC with a correction in error for 4 incorrect reports.
- Pathway C whereby VLN does not identify a ≥80 mm^3^ nodule but the original radiologist did (with or without guideline-based recommendation) would lead to 37/243 (15.2%) of cases reviewed in VNC with correction in error for 7 incorrect reports.

In total, 19 incorrect reports were detected across the three pathways. Five incorrect cases were not modeled, which were identified by VLN and had an incorrect guideline-based recommendation (4) or had VLN algorithm failure (1).

Costs incurred as a direct result of errors in these original reports are summarized in Table 3.

**Table 3:** Additional unit costs per consequence of errors in original radiology reports.

Additional cost item	Unit cost (£)	Frequency
**Discussion in cancer MDT**	51.13	2
**Discussion in respiratory MDT**	51.13	3
**Discussion with specialist radiologist**	78.5	3
**Respiratory consultant review (outpatient follow-up)**	206.85	3
**Respiratory consultant review (referred for initial outpatient appointment)**	271.87	3
**Additional CT scan performed**	121.31	4

Abbreviations: CT = computed tomography, MDT = multidisciplinary team.

Of note, when both VLN and the original radiologist did not identify a lung nodule (115/243, 47.3%) no actionable nodules were identified on ground-truth review.

The incremental cost per additional nodule correctly actioned in pathway A, compared with current practice, was £21.97. Pathways B and C both elicited negative incremental costs, with pathway B generating a cost saving of £40.87 per additional nodule and pathway C a saving of £24.27. At a willingness-to-pay threshold of £22 per additional nodule correctly actioned, all three pathways represent cost-effective strategies when compared to current practice.

## Discussion

This study has shown the incidence of pulmonary nodules in a real-world cohort in a single NHS center in the United Kingdom. The incidence (40.3%) is comparable to previously reported similar cohorts.^13^ We report approximately 10% of cases having an error in the original report leading to incorrect or incomplete recommendation. Extrapolated to a year, this could represent ∼1200 patients with incorrect nodule management in our institution alone.

In an ideal setting, all thoracic CT scans would be interpreted by specialist thoracic radiologists, and in our cohort no errors were identified in cases initially reported by such specialists. However, given current workforce constraints and the limited availability of subspecialist radiologists within the United Kingdom, this model is unlikely to be feasible at scale.^14^ The use of AI to augment the non-specialist review has been touted as a potential to bridge this gap in expertise.^15^

Our study showed a sensitivity and specificity for the identification of actionable pulmonary nodules of >80%. However, this study has importantly not considered VLN in isolation and has modeled triage pathways based upon a combination of original reporting radiologist and automated nodule detection. This combined approach utilizes radiologist visual interpretation and expertise whilst aiding nodule detection using automated nodule identification.

Our study has demonstrated a potential cost saving by triaging cases into a VNC for expert review in cases where a nodule is identified by a first-review radiologist and not by VLN. We hypothesize in these cases the nodules are of more difficult morphology to assess and are therefore more likely to have an error made by non-specialist radiologist. This would include cases of intra-pulmonary lymph nodes and sub-solid nodules. Importantly, the study identified no errors in cases where both the original radiologist and VLN did not identify a nodule.

Argument could be made for review of all cases by a specialist radiologist when either VLN or the original radiologist identifies a lung nodule. This approach was not modeled as would have resulted in 129/243 (53.1%) scans undergoing second review representing too great a burden on existing resources.

The benefit to correct pathway management is beyond that of a pure financial benefit. It is known that patients requiring nodule follow-up can experience a high psychological burden and unnecessary follow-up may reduce this harm (14). Furthermore, outpatient services are under significant workload pressures and unnecessary referrals to respiratory clinics causes delays for patients requiring valid assessment.

The hypothesized pathways demonstrated to have cost saving in this article may facilitate the radical change proposed to the role of multi-disciplinary decision making in cancer care in the United Kingdom.^16^ The study highlighted unnecessary discussion of pulmonary nodules in MDT meetings leading to additional cost and potential delays in decision making. The application of AI-assisted pathway change could avoid such discussions enabling increased time and focus for the cancer MDT to manage more complex cases.

Our study has several limitations, firstly the work is a single center retrospective study performed over one week. There is a risk of case-mix bias, reporting variation which could lead to fluctuation in reporting error. As discussed, no errors were made when the original reporting radiologist was a specialist thoracic radiologist. Further modification of hypothesized pathways may be possible to exclude these cases from analysis; however, this was beyond the scope of the article. The study, however, represents a pragmatic, hypothesized pathway change in a typical secondary care setting the United Kingdom with a moderate size population which reflects many similar institutions both nationally and internationally.

The study included errors made at all stages of reporting from nodule identification to recommended actions. The proposed pathways aim to address all these errors with an AI integrated pathway leading to specialist radiologist review. It is important to note that the application of VLN alone is unlikely to reduce errors in recommended follow-up interval or subsequent nodule management without specialist review.

The study relied upon the ground truth of a single thoracic specialist radiologist; however, this second review was performed by a fellowship trained thoracic radiologist and would be a model for a subspecialist VNC in clinical practice. The analysis performed was following the BTS Nodule Guidance from 2015. There are several other international guidelines that may result in different outcomes from this study, including the Fleishner Society Guidelines 2017 (15). Similarly, due to the study size and restriction to a single clinical location, only limited data was available for economic modeling purposes. Whilst full probabilistic sensitivity analysis was not feasible to quantify uncertainty in cost estimates at this early stage, results indicate potential cost savings across multiple pathway options and warrant further cost-effectiveness evaluation.

## Conclusions

This study demonstrates potential cost savings by integrating automated nodule assessment into routine clinical pathways for patients undergoing thoracic CT scanning. Prospective study is now required to fully assess the cost- and resource-saving benefit of automated triage of lung nodules.

## Supplementary Material

tzag019_Supplementary_Data

## Data Availability

The data underlying this article will be shared on reasonable request to the corresponding author.
