# Supplementary material for: Automated triage to a virtual lung nodule clinic using veye lung nodule volumetry provides a cost saving compared to standard care
Source: BJR Open. 2026 Sep 25;8(1):tzag019. doi: 10.1093/bjro/tzag019 (PMC13654201; doi:10.1093/bjro/tzag019)
Supplement: Supplementary file 1 [file tzag019_supplementary_data.zip › Supp Appendix 1_Economic model cost inputs Changes accepted.docx]

Supplementary Appendix 1 – Economic model cost inputs

| **Resource** | **Cost (£)** | **Source/Rationale** |
| --- | --- | --- |
| CT scan | 121.31 | RD20A (single area, no contrast)^1^ |
| Veye Lung Nodules report | 4.00 | Commercial contract with DeepHealth Ltd |
| Radiologist consultation (per scan read) | 26.17 | £157 per working hr^2^ at 6 scans per hour^3^ |
| Consultant medical | 157 | Cost per working hour (including qualifications)^2^ |
| Consultant surgical | 155 | Cost per working hour (including qualifications)^2^ |
| MDT Respiratory Cancer | 591.87 | (CDMT_OTH other cancer MDT meetings) Resp Med 340^1^ |
| Cost per case discussion in MDT | 19.73 | Based on 30 cases discussed in a 2 hr MDT^1^ |
| Cost per case prepared by expert radiologist for Ca MDT | 15.70 | MDT meeting prep time of 3 hrs for 30 cases at Consultant: Medical hourly rate^2^ |
| Cost per case prepared/administrated by respiratory physician | 15.70 | MDT meeting prep time of 3 hrs for 30 cases at Consultant: Medical hourly rate^2^ |
| Total cost per case discussion in lung MDT | 51.13 | Discussion and prep time combined^1,2^ |
| Additional respiratory consultant review (F/U) | 206.85 | WF01A non-admitted face-to-face, follow-up, consultant led^1^ |
| Additional referral for consultant review | 271.87 | WF01B non-admitted face-to-face, first attendance, consultant led^1^ |
| Additional discussion with chest radiologist | 78.50 | Based on 15 mins radiology consultant and 15 mins physician/surgeon time^2^ |

[1. NHS England » 2024/25 National Cost Collection Data Publication](https://www.england.nhs.uk/publication/2024-25-national-cost-collection-data-publication/)

[2. The unit costs of health and social care 2024 (for publication)_Amended 12 October 2025.pdf](https://kar.kent.ac.uk/109563/1/The%20unit%20costs%20of%20health%20and%20social%20care%202024%20%28for%20publication%29_Amended%2012%20October%202025.pdf)

[3. rcr-publications-radiology-reporting-figures-for-service-planning-2022-november-2022.pdf](https://www.rcr.ac.uk/media/lmojozhq/rcr-publications-radiology-reporting-figures-for-service-planning-2022-november-2022.pdf)
